# A Case of Subdural Empyema Due to Dental Pathogen

**DOI:** 10.7759/cureus.43666

**Published:** 2023-08-17

**Authors:** Gabriela Portilla-Skerrett, Pedro Rivera-Ramírez De Arellano, Carmarie Santiago-Ortolaza, Dev Boodoosingh-Casiano

**Affiliations:** 1 Internal Medicine, Hospital Episcopal San Lucas, Ponce, PRI; 2 Internal Medicine, Centro Medico Episcopal San Lucas, Ponce, PRI

**Keywords:** acute neurological deficit, burr holes procedure, otorhinology infection, dental caries, subdural empyema

## Abstract

Subdural empyema is a rare but serious infection in the brain. Several etiologies and pathological mechanisms have been described. The team reports a case of subdural empyema due to dental pathogens, of which a limited number of cases have been reported. Radiological findings and medical management of this case are reviewed since prompt intervention reduces not only mortality and morbidity but also complications including sepsis, cranial osteomyelitis, and residual neurological deficit, among others.

## Introduction

A subdural empyema (SE) is a collection of pus between the dura and the arachnoid membrane. SE is a serious condition with a high morbidity and mortality rate that requires prompt medical and neurosurgical intervention [1]. It can also be classified as a brain abscess. According to the literature, 40%-80% of SE cases have otorhinology infection, 20% of cases occur following head trauma or cranial surgical procedures, and others rarely have a blood infection [1-3]. The most common organisms found in abscesses/empyemas are anaerobes such as *Streptococci*, *Staphylococcus*, and *Haemophilus *​​​​*influenzae*. Predisposition factors for SE include head trauma, prior cranial surgery, subdural hematoma, and poorly treated ear, mouth, or sinus infections. Symptoms may vary between patients. The most common symptoms are fever, headache, and neurological deficits. Additionally, they may present with episodes of nausea, vomiting, mental status changes, signs of meningeal irritation, and an increase in intracranial pressure. SE requires prompt management because it can lead to complications such as acute neurological deficits, seizures, sepsis/shock, cerebral edema, and cerebral osteomyelitis, among others. 

Early recognition, intravenous antibiotics, and timely surgical intervention significantly decrease morbidity and mortality [3,4]. Through this case report, the team intends to educate and create awareness of SE, an infection that warrants early medical and surgical intervention.

## Case presentation

A 30-year-old male with a past medical history of hypertension, chronic sinusitis, and childhood bronchial asthma arrived at the Emergency Room (ER) with blurry vision, worsening headache, and weakness in his left lower extremity. Within one month, the patient developed fever episodes, blurry vision, frontal headache, generalized weakness, and pronounced numbness in his lower left extremity. Two weeks prior to ER arrival, the patient had been diagnosed with COVID-19 and treated symptomatically. Upon initial evaluation, the physical examination revealed a Glasgow Coma Scale (GCS) of 13/15. The patient was awake, alert and oriented. His cranial nerves were grossly intact and showed isochoric pupils, weakness in the lower left extremity 4/5, and decreased pain sensation. Vitals signs were remarkable for a temperature of 101.1 °F. Initial laboratories showed leukocytosis, an elevated lactic acid level, and a urinalysis positive for urinary tract infection.

**Figure 1:**
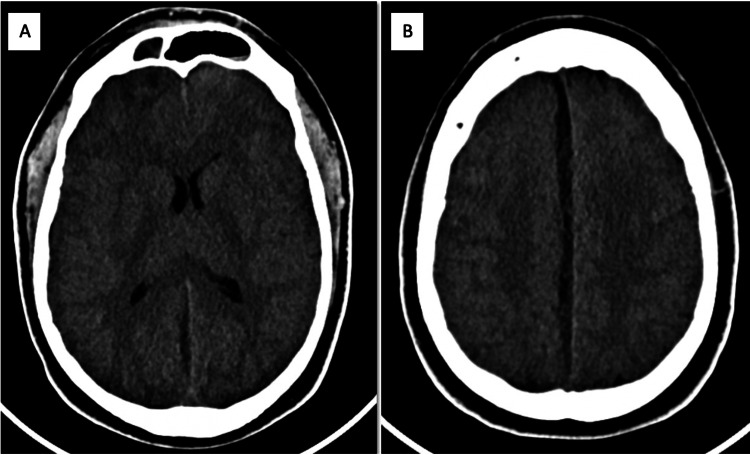
Initial CT without contrast showing right frontal hypodense fluid collection (A) and hypodense subdural collection in falx consistent with hematoma vs empyema (B)

A head computed tomography scan was performed and showed subdural hematoma vs empyema (Figures 1A, 1B), for which intravenous antibiotics were administered and both the neurosurgeon and the infectologist were consulted. The patient was admitted to the hospital due to an acute neurological deficit. Two days after admission, the patient presented with neurological decline and septic parameters. The patient was transferred to the intensive care unit and antibiotics were optimized due to bacteremia by *S. anginosus*. A few hours later, the patient was intubated to protect his airway and taken to the operating room by the neurosurgeon for a burr holes procedure. During the surgery, purulent secretions were collected and sent for microbiological identification but showed no organism.

**Figure 2:**
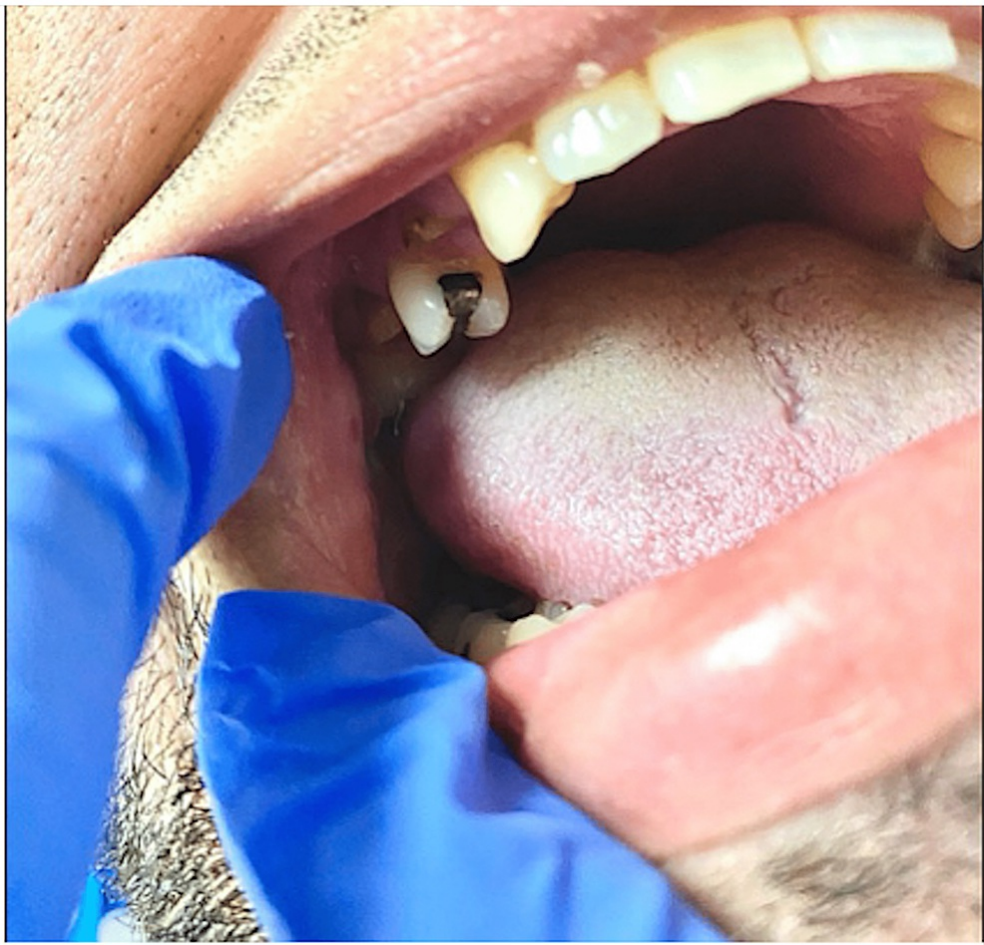
Patient’s right upper and lower molar cavities and missing denture

**Figure 3:**
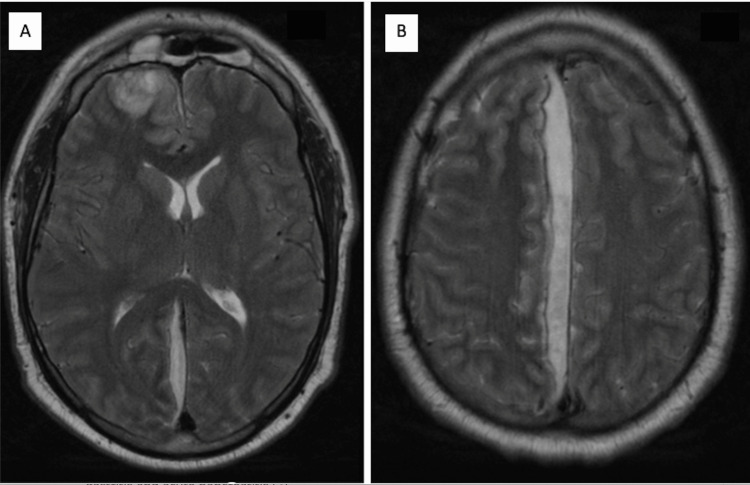
Brain MRI with IV contrast showing right frontal lobe lesion, which can be infectious and/or neoplastic in nature (A), and subdural fluid collection along the falx towards the right midline extending into the right tentorium (B)

Upon further evaluation of possible etiologies, her mother informed the hospital’s Internal Medicine team of multiple chronic tooth infections (Figure 2) that had not been previously treated. The patient's brain MRI (Figures 3A, 3B) revealed multiple brain abscesses and SE, correlating with the patient’s physical findings, and the blood culture organism *S. anginosus*. For this reason, a dentist was consulted, and the decayed molars were surgically removed. After a prolonged course of IV antibiotics, surgery, and physical therapy, the patient recovered satisfactorily with complete neurological improvement and resolution of symptoms without major complications. Days later, the patient was discharged home for an outpatient follow-up with the neurosurgeon.

## Discussion

SE is a rare condition and diagnosis can be challenging because patients can present various symptoms [5]. The patient discussed in this report suffered from fever, headache, and acute neurological changes. Per the literature, half of the patients with SE present these symptoms. The image of choice for SE is a brain MRI with intravenous contrast (sensitivity 93%), even though a head CT scan is more readily available and less expensive than MRI. Further workup includes complete blood cell count, erythrocyte sedimentation rate, C-reactive protein (acute phase reactants), and blood culture. Once SE is diagnosed with imaging, immediate management is imperative with IV antibiotics and neurosurgical intervention.

It has been shown that SE has multiple etiologies. Nevertheless, the team’s patient exhibited a clear cause for SE that, as per literature, could have been prevented with adequate routine dental and oral hygiene care and early removal of the abscess, if the case [6]. *S. anginosus*, found in the patient’s bloodstream, is a subgroup of the *S. viridans,* which is also found in the normal oral flora and the gastrointestinal tract. *S. anginosus* tends to form central nervous system abscesses, empyema, and systemic infections. SE may arise due to bacteremia from oral/dental or gastrointestinal tract infections [7-9]. Oral cavity pathogens may reach the brain via 1) systemic hematogenous bacteremia (after an invasive oral procedure or even spontaneously), 2) direct contiguity, or 3) direct venous drainage [7]. The spread of bacteremia typically forms multiple lesions. For this reason, the team suspects *S. anginosus* bacteremia was the cause of the patient’s intracranial infection. 

SE requires medical and surgical intervention, in addition to infectiology and neurosurgery services. Surgical management includes burr holes and/or craniotomy; craniotomy usually offers better results and fewer recurrences [1]. In the discussed patient, a burr holes procedure was performed, as the brain was severely edematous. A sample of the abscess/empyema was taken to guide the antibiotic therapy; however, no organism grew in the medium. The literature states that seven percent to 53 percent of patients’ cultures are negative [3,4]. Sterile empyema might be due to early administration of antibiotics or improper use of culture medium. Still, an empiric regimen against *staphylococcus*, *streptococcus*, anaerobes, and gram-negative bacilli must be provided (metronidazole and ceftriaxone or cefotaxime which can enter the cerebrospinal fluid). Furthermore, anti-epileptic drugs should be given in view of the high incidence of perioperative seizures and steroids to decrease inflammation and cerebral edema [1,3,5]. As seen in our case, if SE is due to tooth infection, experts believe dental extraction may be required in addition to antibiotics [10]. Moreover, the antibiotics course for SE ranges from three to eight weeks and should include vancomycin or nafcillin, metronidazole, and ceftriaxone or cefepime/ceftazidime for pseudomonas coverage [11,12]. Therapy should be determined by blood work, surgical drainage, bacteriology, radiographic findings, and clinical response. 

Management for this condition should consist of a focused evaluation including a thorough physical exam, imaging, broad-spectrum antibiotics, and prompt surgery. Early management significantly decreases morbidity, mortality, and the severity of neurological sequelae. Moreover, patient prognosis depends on the preoperative level of consciousness, the timing of intervention, and the aggressiveness of the management. Thus, in a patient with a history of oral infections presenting with headache, fever, and neurological changes, intracranial infections should be highly suspected.

## Conclusions

SE can present itself in several ways, fever and headache being the most common. It has significant morbidity and mortality. The team’s goal is to educate and create awareness about the medical and surgical emergent management that is warranted for SE. Moreover, if a patient presents with* S. anginosus *bacteremia, one should further investigate underlying abscesses and treat them accordingly. This case is a prime example of how prompt identification and treatment of SE can decrease mortality and neurological disability.
